# Young Adults’ New Cognitive Formations and the Feeling of Becoming an Adult

**DOI:** 10.1192/j.eurpsy.2023.1551

**Published:** 2023-07-19

**Authors:** O. V. Markish, S. M. Churbanova, O. B. Chesnokova

**Affiliations:** 1 Department of Developmental Psychology, Faculty of Psychology, Lomonosov Moscow State University, Moscow, Russian Federation

## Abstract

**Introduction:**

In adolescence, the person transitions from the reality of childhood, where he is mostly dependent on his parents, to the reality of adult life, which necessitates the acquisition of adult role systems, established autonomy, and ability to accept responsibility. The most important feeling during that time is that of maturation into adulthood.

**Objectives:**

This study was designed to investigate the impact of new cognitive formations on the maturation of young adults’ self-consciousness from the perspective of a subjective evaluation of the experience of becoming an adult, particularly its cognitive component.

**Methods:**

The study was based on Akimova’s Adult Practical Thinking Scale (Akimova et al., 2008), Zack’s Theoretical Thinking Scale (Zack, 2010), Personal Differential Test (Bazhin & Etkind, 1983), Szustrowa’s Egocentric Associations Scale with content analysis applied (Szustrowa, 1976), The Feeling of Becoming an Adult Expression Scale (Andriushchenko et al., 2014) and included 64 participants aged 18-22 years. The approbation group had 12 participants and the core group had 52 participants.

**Results:**

The IBM SPSS 22 statistical rank correlation analysis provides support for: negative moderate correlation between (1) the reflective type of theoretical thinking and personal egocentrism (rs=-.31; p=.024), (2) adult practical thinking level and awareness of a new position in adolescents-adults relations self-consciousness component (rs=-.28, p=.048); positive moderate correlation between (1) social intelligence and the feeling of becoming an adult expression degree (rs=.39; p=.004), (2) orientation to autonomous educational and intellectual activity (self-consciousness component) and subjective attitude towards oneself and other people (rs=.29, p=.04), orientation to autonomous educational/intellectual activity and Egocentrism Index (rs=.37, p=.007).

**Conclusions:**

According to the study’s findings, there is a strong correlation between young adults’ cognitive traits (such as content-related reflection for formal operational thinking and social intelligence), their expression of the feeling of becoming an adult, and some aspects of self-consciousness. It indicates that the feelings of becoming an adult manifest themselves as an orientation to autonomous educational and intellectual activity and new forms of cooperation with adults that develop step by step through time, but not as a process of individualization based on egocentric attitudes.

**Disclosure of Interest:**

None Declared

